# Multi-kingdom microbial signatures in excess body weight colorectal cancer based on global metagenomic analysis

**DOI:** 10.1038/s42003-023-05714-0

**Published:** 2024-01-05

**Authors:** Xinyue Zhu, Pingping Xu, Ruixin Zhu, Wenxing Gao, Wenjing Yin, Ping Lan, Lixin Zhu, Na Jiao

**Affiliations:** 1 Putuo People’s Hospital, School of Life Sciences and Technology, Tongji University, Shanghai, PR China; 2 Department of Colorectal Surgery, Zhongshan Hospital, Fudan University, Shanghai, PR China; 3 Guangdong Institute of Gastroenterology; Guangdong Provincial Key Laboratory of Colorectal and Pelvic Floor Diseases; Biomedical Innovation Center, Sun Yat-Sen University, Guangzhou, PR China; 4 Department of General Surgery, The Sixth Affiliated Hospital of Sun Yat-Sen University, Guangzhou, PR China; 5 National Clinical Research Center for Child Health, the Children’s Hospital, Zhejiang University School of Medicine, Hangzhou, Zhejiang PR China

**Keywords:** Cancer screening, Clinical microbiology

## Abstract

Excess body weight (EBW) increases the risk of colorectal cancer (CRC) and is linked to lower colonoscopy compliance. Here, we extensively analyzed 981 metagenome samples from multiple cohorts to pinpoint the specific microbial signatures and their potential capability distinguishing EBW patients with CRC. The gut microbiome displayed considerable variations between EBW and lean CRC. We identify 44 and 37 distinct multi-kingdom microbial species differentiating CRC and controls in EBW and lean populations, respectively. Unique bacterial-fungal associations are also observed between EBW-CRC and lean-CRC. Our analysis revealed specific microbial functions in EBW-CRC, including D-Arginine and D-ornithine metabolism, and lipopolysaccharide biosynthesis. The best-performing classifier for EBW-CRC, comprising 12 bacterial and three fungal species, achieved an AUROC of 0.90, which was robustly validated across three independent cohorts (AUROC = 0.96, 0.94, and 0.80). Pathogenic microbial species, *Anaerobutyricum hallii*, *Clostridioides difficile* and *Fusobacterium nucleatum*, are EBW-CRC specific signatures. This work unearths the specific multi-kingdom microbial signatures for EBW-CRC and lean CRC, which may contribute to precision diagnosis and treatment of CRC.

## Abstract (web summary)

Metagenomic analysis reveals distinctive multi-kingdom species, functional signatures and bacteria-fungi interactions pattern between excess body weight CRC and lean CRC in gut microbiome, which contribute to precision medicine of CRC.

## Introduction

Colorectal cancer (CRC) is the third most common malignancy worldwide, accounting for 9.4% cancer-related death^1,2^. Besides genetic factors, modern lifestyles, such as the intake of a high-fat diet and lack of physical activity contribute to the increasing incidence of CRC^3–5^. Epidemiological data suggest a positive relationship between excess body weight and CRC incidence, whose relative risk attributable to excess body mass index (BMI) is 1.24 for men overall^6^, ranging between 1.04 and 1.27 across countries^7^. Moreover, obesity in early adulthood is also strongly associated with an increased risk of CRC^7,8^.

Lines of evidence support close associations between dysregulated microbiota and the development of CRC, emphasizing the roles of altered microbial composition, function and microbe-derived metabolites^9–12^. In addition, gut microbiota is a critical player in contributing to the onset and development of obesity^13–15^. A common microbial etiology for CRC and obesity could be mediated by chronic low-grade inflammation, a hallmark for both obesity and CRC^16–18^. Increased abundance of *Fusobacterium nucleatum* and enterotoxigenic *Bacteroides fragilis* could activate the nuclear factor kappa B pathway, thereby generating a pro-inflammatory environment conducive to colorectal neoplasia progression^19,20^. Another shared link between obesity and CRC is that high-fat diet increases the microbes-derived lysophosphatidic acid, impairs gut barrier and drives colorectal tumorigenesis^12^. However, characteristics of microbiome in overweight or obesity-related CRC remain elusive.

Whereas numerous CRC screening programs have been launched worldwide, compliance with colonoscopy, the current gold standard for screening, is relatively lower in individuals with excess weight than the normal-weight target population^21^. Besides, the quality of bowel preparation in obese individuals is found to be inferior to that in normal BMI controls^22^. Gut microbiota has emerged as a promising, non-invasive, and easily accessible biomarker for CRC^23^. Analyses that integrate characteristics of multiple cohorts contribute to identifying universal and robust microbial markers. Our studies, along with others, have established several candidate panels of microbial biomarkers to effectively distinguish patients with CRC, colorectal adenoma, or other microbes-related diseases from controls based on 16 S rRNA or whole metagenome sequencing data^24–31^. Notably, besides bacteria, microbial multi-kingdom signatures including fungi, archaea and viruses, have been associated with CRC^28,32–35^. Furthermore, the combination of multi-kingdom microbial biomarkers achieved higher capabilities in detecting patients with CRC^28,35^. Nevertheless, it remains to be elucidated whether microbial signatures with capability to discriminate overweight or lean CRC patients.

Therefore, we performed a comprehensive multi-center study, examining unique microbial signatures between excess body weight CRC (EBW-CRC) and lean-related CRC (lean-CRC) with 981 samples from eight diverse cohorts spanning various geographical and cultural origins. The distinctive multi-kingdom microbial signatures, interkingdom associations and functional alterations were examined in both EBW-CRC and lean-CRC patients. Based on these specific microbial signatures, we further identified and validated two distinct microbial biomarker panels, each exhibiting robust capability for distinguishing EBW-CRC and lean-CRC, with three independent cohorts.

## Result

### Multi-cohort CRC metagenomic data and annotation of multi-kingdom taxonomic and functional profiles

We collected whole metagenomics data from 981 human fecal samples from seven publicly available CRC cohorts and one in-house CRC cohort, which included 209 samples from EBW-CRC, 179 samples from excess body weight controls (EBW-CTR), 276 samples from lean-CRC patients and 317 samples from lean controls (lean-CTR) (Table 1 and Supplementary Data 1). To minimize heterogeneity, we reanalyzed the whole metagenomics data using a uniform bioinformatics pipeline to obtain multi-kingdom abundance profiles, covering bacterium, fungus, archaea, and virus, as well as functional profiles individually for each cohort. To better characterize universal and robust gut microbial signatures for EBW-CRC and lean-CRC, samples from Austria, China, France, Germany, and Italy with broad geographical and cultural backgrounds were selected as discovery datasets for constructing diagnostic models, while the remaining cohorts were used for model validation, including two China cohorts, Chongqing and Shanghai (in-house data), and USA cohort.

**Table 1:** Demographic information across all cohorts.

Cohort			EBW (excess body weight)^a^	lean (normal body weight)^b^			Data source
			CRC	CTR	CRC	CTR	
			*N* = 209	*N* = 179	*N* = 276	*N* = 317	
Discovery Cohorts	AUS	Number	30	42	16	21	Feng et al.^46^.
		Age^c^	67.17 ± 10.61	68.12 ± 5.66	66.88 ± 11.82	64.95 ± 7.29	
		BMI^c^	28.61 ± 1.99	30.06 ± 1.55	22.56 ± 2.05	22.6 ± 0.56	
		Gender^d^	9/21	15/27	9/7	11/10	
	CHI	Number	25	10	48	44	Yu et al.^36^.
		Age^c^	65.44 ± 9.89	60.4 ± 5.87	66.4 ± 11.14	62.07 ± 5.64	
		BMI^c^	27.36 ± 1.70	28.07 ± 3.20	22.23 ± 2.15	22.42 ± 1.61	
		Gender^d^	13/12	4/6	13/35	17/27	
	FRA	Number	54	32	35	32	Zeller et al.^59^.
		Age^c^	67.22 ± 9.8	62.41 ± 7.76	66.42 ± 12.25	58.87 ± 14.24	
		BMI^c^	29.02 ± 4.47	27.14 ± 2.42	21.76 ± 2.46	22.32 ± 1.63	
		Gender^d^	10/17	14/15	13/11	18/12	
	GER	Number	12	27	10	33	Wirbel et al.^25^.
		Age^c^	62.38 ± 11.88	56.03 ± 11.89	65.43 ± 14.02	55.91 ± 12.54	
		BMI^c^	28.44 ± 2.64	27.59 ± 2.34	22 ± 2.38	22.61 ± 1.75	
		Gender^d^	14/25	11/19	10/11	17/18	
	ITA	Number	14	12	14	10	Thomas et al.^26^.
		Age^c^	69.21 ± 7.72	69 ± 7.1	73.43 ± 8.71	66.4 ± 6.75	
		BMI^c^	28.79 ± 3.66	27.58 ± 3.00	22.64 ± 1.39	22.6 ± 1.65	
		Gender^d^	2/12	5/7	4/10	4/6	
Validation Cohorts	CHI_CQ	Number	28	18	70	77	Yang et al.^60^.
		Age^c^	57.29 ± 11.41	45.06 ± 11.2	54.17 ± 10.79	42.43 ± 10.37	
		BMI^c^	27.02 ± 1.44	25.86 ± 0.88	21.99 ± 1.86	22.41 ± 1.37	
		Gender^d^	8/20	9/9	22/48	37/40	
	CHI_SH	Number	24	18	56	68	Liu et al.^28^.
		Age^c^	58.5 ± 8.37	60.17 ± 7.49	58.48 ± 10.71	58.18 ± 8.77	
		BMI^c^	27.45 ± 1.84	27.29 ± 1.96	22.05 ± 2.07	21.89 ± 1.96	
		Gender^d^	10/14	7/11	22/34	27/41	
	USA	Number	22	20	27	32	Vogtmann et al.^61^.
		Age^c^	61.5±8.14	61.6±10.03	60.52±16.69	61±11.76	
		BMI^c^	28.57±3.17	29.6±3.59	21.89±2.09	22.68±1.80	
		Gender^d^	7/15	7/13	6/21	8/24	

^a^Subjects are overweight (BMI $\ge$25 kg/m^2^) or obese (BMI $\ge$30 kg/m^2^);

^b^Subjects with normal BMI (BMI < 25 kg/m^2^);

^c^Mean ± sd;

^d^Female/Male.

### Comprehensive characterization of taxonomic and functional signatures in EBW-CRC and lean-CRC

#### Taxonomic alteration patterns

**Fig. 1:**
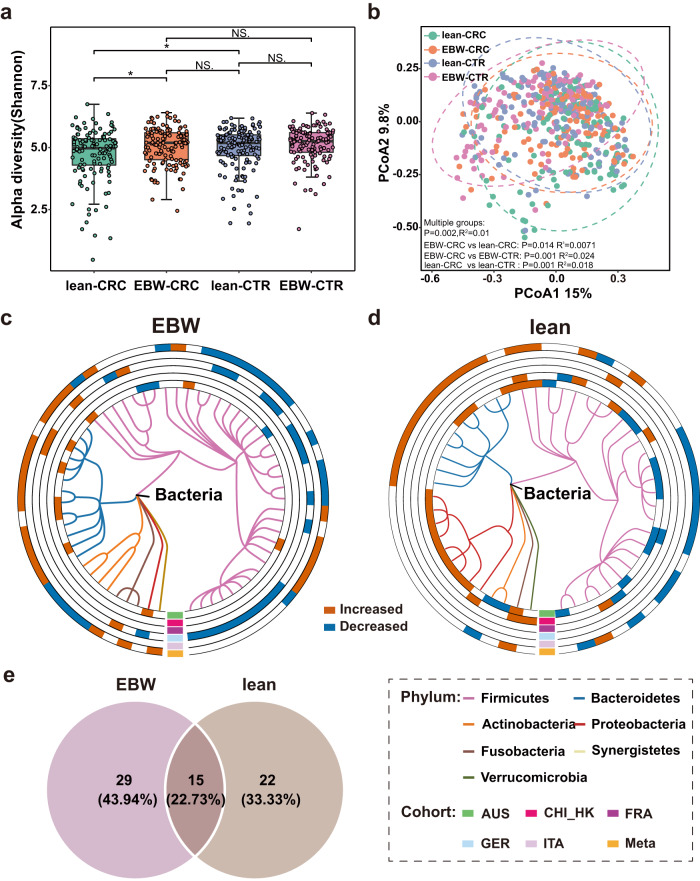
Diversities of fecal microbiota, differential bacterial species and venn diagram of all differential species. **a** Boxplot of alpha diversities measured by Shannon index of lean-CRC, EBW-CRC, lean-CTR and EBW-CTR groups. (*n* = 123, 135, 140 and 123 for lean-CRC, EBW-CRC, lean-CTR and EBW-CTR, respectively). Statistical differences were evaluated by Wilcoxon rank sum test. **b** Beta diversities of the discovery cohorts were assessed by principal coordinate analysis (PCoA) based on Bray-Curtis dissimilarity. *P* values and R-square values of beta diversity based on Bray–Curtis distance were calculated with PERMANOVA by 999 permutations (two-sided test). **c**, **d** Phylogenetic tree of the differential bacterial species in EBW-CRC (**c**, 60 species) and lean-CRC (**d**, 50 species). The outer circles are marked as significantly differential species (FDR-corrected *P* < 0.05) in each cohort and in the meta-analysis (meta-ring) with red for increased abundance and blue for decreased abundance. **e** Venn diagram of all differential species in EBW- and lean-CRC identified from the meta-analysis of the discovery cohorts.

Though alpha diversities were similar between EBW-CRC and EBW-CTR, significantly decreased alpha diversities were observed in lean-CRC, compared with lean-CTR (*P* < 0.05, Fig. 1a). Notably, we found that alpha diversities of EBW-CRC group were higher than that of lean-CRC group (*P* < 0.05, Fig. 1a). Beta diversity also varied significantly between EBW-CRC and lean-CRC (PERMANOVA, *P* = 0.014, ${R}^{2}$ = 0.0071, Fig. 1b). Furthermore, the microbial compositional distribution was significantly distinct between CRC patients and controls in both EBW (PERMANOVA, *P* = 0.001, ${R}^{2}$ = 0.024) and lean populations (PERMANOVA, *P* = 0.001, ${R}^{2}$ = 0.018).

Consistent with our previous studies, heterogeneous microbial alternations were observed among geographically distinct cohorts (Fig. 1 and Supplementary Fig. 1). To identify essential differential species across cohorts, integrated analyses with MMUPHin, which considered the bias caused by potential confounders, were performed and identified a total of 44 multi-kingdom species with differential abundance between EBW-CRC and EBW-CTR. Among these, 22 out of 40 differential bacterial species were enriched in CRC, such as *Akkermansia muciniphila*, *Alistipes indistinctus*, *Anaerotruncus colihominis, B. fragilis*, while the other 18 species, including *Adlercreutzia equolifaciens*, *Bifidobacterium adolescentis* and *Butyrivibrio fibrisolvens*, were decreased in CRC (Fig. 1c and Supplementary Data 2) A majority of differential bacterial signatures were members from phylum Firmicutes. Meanwhile, three fungal species, namely *Aspergillus rambellii*, *Metarhizium acridum* and *Stemphylium lycopersici*, showed increased abundance in CRC, while only *Colletotrichum orbiculare*, displayed a decrease (Supplementary Fig. 1a and Supplementary Data 2). All these differentially abundant fungi were members of the phylum Ascomycota.

Similarly, integrated-analyses determined 37 differential microbial species across all four kingdoms between lean-CRC and lean-CTR. Among these differential bacterial species, decreased abundances in CRC were observed for 13 species, including *Pseudobutyrivibrio xylanivorans*, *Blautia liquoris* and *Bifidobacterium breve*, while 15 bacterial species, including *B. fragilis*, *Gemella morbilloru*m and *Parvimonas micra*, exhibited increased abundances in CRC (Fig. 1d and Supplementary Data 3). For differential fungal species, the abundances of six out of eight species, such as *A. rambellii* and *Erysiphe pulchra*, were increased in CRC (Supplementary Fig. 1b and Supplementary Data 3). Only two differential fungal species including *C. orbiculare* and *Rhizophagus clarus* decreased in lean-CRC. Additionally, only one differential viral species, *crAssphage cr4_1*, was identified as differing between lean-CRC and lean-CTR (Supplementary Data 3).

Comparing the differential taxonomic signatures in the lean group with those in the EBW group, 15 differential species were common for both groups (Fig. 1e and Supplementary Data 4), accounting for 22.73% of total differential species. This sets includes the previously reported CRC diagnostic biomarkers^24,36,37^, such as *P. micra, Porphyromonas asaccharolytica*, *Prevotella intermedia* and *F. nucleatum* as well as pathogenetic species^38–40^ *B. fragilis* and *G. morbillorum*. Besides, *A. rambellii* was CRC-associated fungal species enriched in both EBW and lean individuals^35^. Importantly, we observed a large proportion of differential signatures being specific for EBW (29, 43.94%, Supplementary Data 5) or lean individuals (22, 33.33%, Supplementary Data 6). In EBW-CRC populations, *Coprococcus comes* and *Clostridioides difficile* were decreased, while *Parabacteroides distasonis* and *Flavonifractor plautii* were increased, and such pattern was not observed in lean-CRC populations. Contrarily, in lean-CRC but not in EBW-CRC patients, *Dialister pneumosintes* and *Streptococcus oralis* were increased, while *Faecalibacterium prausnitzii* and *Streptococcus salivarius* were decreased. It’s noteworthy that *F. prausnitzii* in collaboraion with *Carnobacterium maltaromaticum*, helps to convert 7-dehydrocholesterol into vitamin D, ultimately activating the host vitamin D receptor (VDR) to suppress CRC^41^.

#### Microbial ecological alterations

To characterize the microbial ecological patterns in different populations, we next examined the co-abundance associations among multi-kingdom differential species in both EBW and lean individuals. Complex patterns of associations among differential species were observed in both EBW-CRC group (44 species and 429 associations, Supplementary Fig. 2a and Supplementary Data 7) and EBW-CTR group (44 species and 512 associations, Supplementary Fig. 2b and Supplementary Data 8). Meanwhile, there were 204 co-abundance associations among 37 species in the lean-CRC group (Supplementary Fig. 2c and Supplementary Data 9), with a sparser pattern than that of the EBW-CRC group. The lean-CTR group also exhibited considerably fewer associations featuring 232 associations among 36 species (Supplementary Fig. 2d and Supplementary Data 10). Analysis of interkingdom associations revealed a remarkable increase of positive association numbers in EBW-CRC compared with lean-CRC (Chi-square test, *P* = 4.45 × 10^-12^). Moreover, among these associations, the EBW-CRC exhibited a unique symbiotic relationship not observed in lean-CRC, characterized by exclusive positive interactions between fungal species *A. rambellii* and bacterial species, including *B. fragilis*, *Fusobacterium gonidiaformans*, *F. nucleatum*, and *G. morbillorum*. However, in lean individuals, *A. rambellii* only showed a negative correlation with various *Faecalibacterium* species (*F. prausnitzii*, *F. sp. I3389*, *F. sp. I3333, F. sp. I4179, F. sp. I4384*) in CRC. Another outstanding observation was that, the positive association between *A. rambellii* and *F. nucleatum* was only observed in EBW-CRC rather than in lean-CRC.

**Fig. 2:**
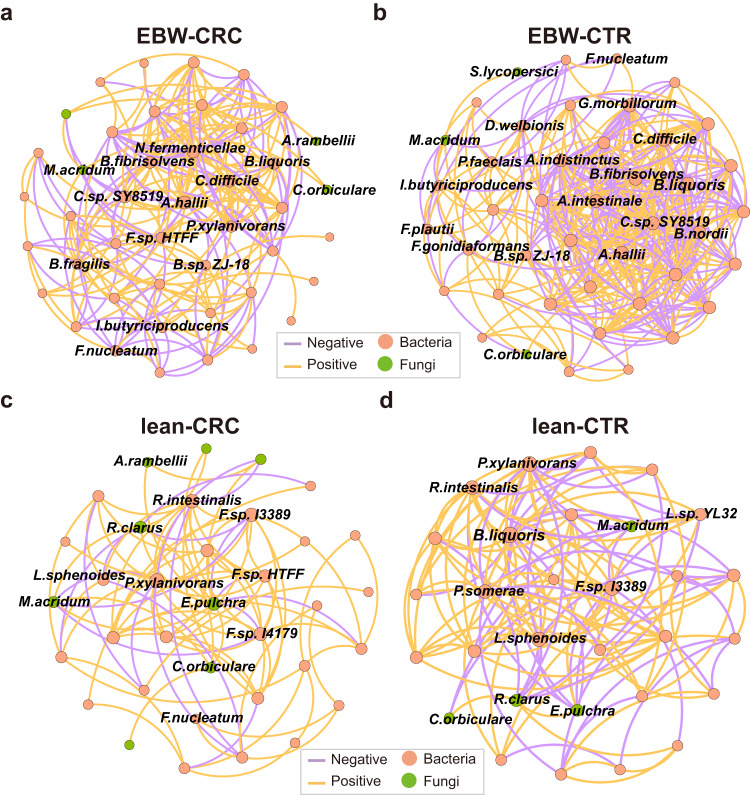
Microbial co-abundance networks of multiple kingdom species in EBW-CRC, EBW-CTR, lean-CRC and lean-CTR. Co-abundance networks for EBW-CRC (**a**, 44 species and 218 associations), EBW-CTR (**b**, 43 species and 349 associations), lean-CRC (**c**, 35 species and 95 association) and lean-CTR (**d**, 30 species and 128 associations). Strong correlations with absolute value of correlation coefficients above 0.3 and a significant cut-off of FDR-corrected *P* < 0.05 were plotted. Colors of the nodes represent different kingdoms. Edge colors indicate positive or negative correlation.

We subsequently focused on the strong associations ( | correlation coefficient | > 0.3). Surprisingly, in the EBW groups, the amount of strong microbial associations in CRC (44 species and 218 associations, Fig. 2a) was much sparser than that in controls (43 species and 349 associations, Fig. 2b). Furthermore, in the CRC co-abundance network, most associations were positive, with much fewer being negative associations (34.86%, 76 associations) compared to that in the controls (CTR) network (44.99%, 157 associations), suggesting more competitive pressure in CRC community. Some of the strong co-abundance associations, such as those between *S. lycopersici* and *Faecalibacterium sp. HTFF*, between *M. acridum* and *B. fragilis*, and between *M. acridum* and *C. difficile*, appeared in CRC but not in CTR. Similarly, some strong associations in the CTR network were missing in CRC network, such as the associations between *Bacteroides nordii* and *P. intermedia*, between *Anaerobutyricum hallii* and *B. nordii*, and between *C. orbiculare* and *F. plautii*. In lean groups, 95 strong associations were identified among 35 species in CRC (Fig. 2c), which is also sparser than that in control groups (30 species and 128 associations, Fig. 2d).

These co-abundance networks clearly showed that EBW-CRC community was more complex than lean-CRC community. Furthermore, the EBW-CRC network displayed a reduced proportion of positive correlations compared to the lean-CRC network (Chi-square test, *P* < 2.2 × 10^-16^), and the EBW-CRC network also exhibited fewer interkingdom associations than the lean-CRC network. Nevertheless, multiple positive interkingdom associations were exclusively observed in the EBW-CRC network, including associations between *M. acridum* and *B. fragilis, Bacteroides sp. ZJ-18*. In contrast, the lean-CRC network also had unique interkingdom associations, including fungal species *E. pulchra*, *Pyrenophora tritici-repentis* and *R. clarus*. Key species varied in different microbial communities, with key species *A. hallii*, *Clostridium sp. SY8519*, *B. fibrisolvens, C. difficile* and *Novisyntrophococcus fermenticellae* in the EBW-CRC community, and key species *P. xylanivorans*, *F. sp. I3389*, *Roseburia intestinalis*, *F. sp. I4179* and *E. pulchra* in the lean-CRC community.

#### Differential microbial functions between EBW-CRC and lean-CRC

**Fig. 3:**
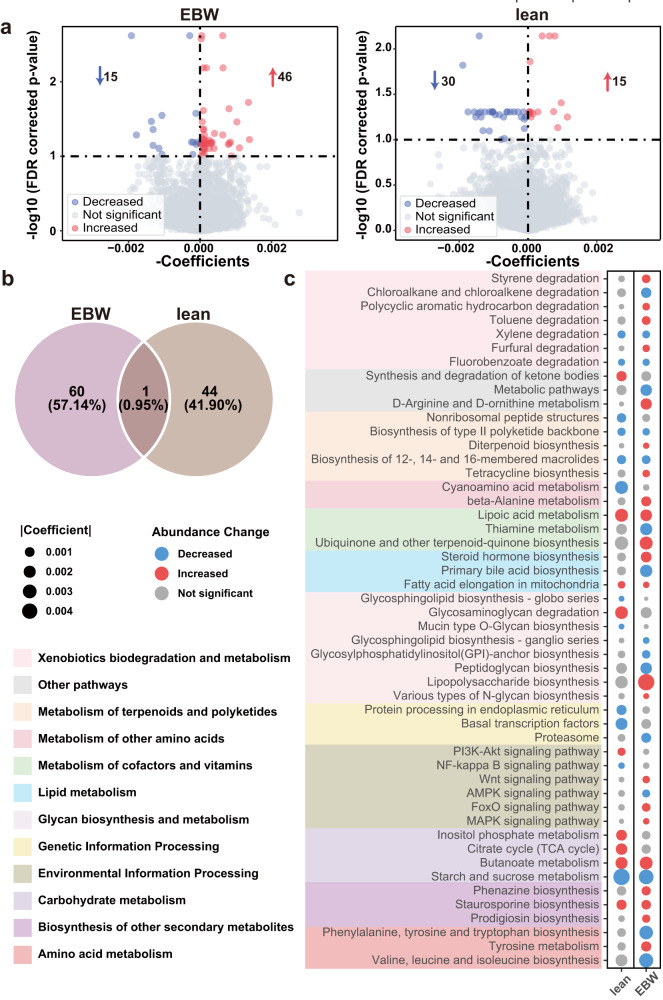
Functional alteration in gut microbiome of excess body weight and lean CRC patients. **a** Volcano plots of differential KO genes in excess body weight (EBW) and lean CRC patients compared to CTR. **b** Venn diagram of the differential KO genes in the EBW and the lean groups. **c** Bubble chart shows the important part of significantly differential functional pathways in the EBW and the lean groups, with red dot indicating increased abundance, and blue dot decreased abundance. The size of dots indicates the degree of the alteration.

Differential analysis at the gene level identified 61 differential KEGG orthology (KO) genes between EBW-CRC and EBW-CTR groups, with 15 KO genes being decreased and 46 KO genes being elevated in patients with CRC (Fig. 3a and Supplementary Data 11). On the other hand, 30 KO genes with reduced abundances and 15 KO genes with increased abundances were identified in lean-CRC in comparison to lean-CTR (Fig. 3a and Supplementary Data 12). The differential KO genes were considerably distinct between EBW-CRC and lean-CRC, with only one common differential KO gene, *NDUFA8*(K03952) (Fig. 3b and Supplementary Data 13). While KO genes involved in metabolism, *cysA* (K02045) and *gctB* (K01040), were EBW-CRC specific differential genes (Supplementary Data 14), differential genes *gudB* (K00260) and *metH* (K00548) were specific for the lean group (Supplementary Data 15).

At the pathway level, 47 differential pathways were identified in the EBW group with 24 increased pathways, such as toluene degradation, tyrosine metabolism and phenazine biosynthesis, and 23 decreased pathways, including valine, leucine and isoleucine biosynthesis, starch and sucrose metabolism (Fig. 3c and Supplementary Data 16). Conversely, in the lean group, 35 differential pathways were identified consisting of 17 increased pathways, like synthesis and degradation of ketone bodies, inositol phosphate metabolism, and 18 decreased pathways, such as starch and sucrose metabolism, and cyanoamino acid metabolism (Fig. 3c and Supplementary Data 17). Notably, both butanoate metabolism and lipoic acid metabolism showed increased relative abundances in CRC compared to controls for EBW and lean groups. This observation aligns with findings from our previous study^28^, suggesting these alterations may be essential to CRC. Conversely, our prior study indicated enhanced metabolic potentials of D-Arginine and D-ornithine metabolism in patients with CRC^28^, while here we found that this pathway was specifically elevated in EBW-CRC. Besides, pathways related to xenobiotics biodegradation and metabolism, such as toluene, polycyclic aromatic hydrocarbon and styrene degradation, were uniquely elevated in the EBW group, while valine, leucine and isoleucine biosynthesis specifically decreased in the EBW group. Meanwhile, inositol phosphate metabolism, and synthesis and degradation of ketone bodies were differential pathways specific for lean-CRC. Collectively, our results underscore distinct microbial functional features between excess body weight and lean CRC patients.

### Identification and validation of multi-kingdom microbial signatures for CRC

Given the distinct microbial signatures observed at both taxonomic and functional levels in EBW-CRC and lean-CRC, there is potential for microbial signatures to serve as precise indicators for detecting EBW-CRC and lean-CRC. To this end, we determined multidimensional signatures and assessed the classification efficacy via *xMarkerFinder*.

#### Identification of EBW-CRC associated microbial signatures

Firstly, we identified single kingdom taxonomic signatures for excess body weight individuals, and found that bacterial signatures outperformed signatures of other kingdoms. The classifier based on 13 bacterial species achieved an average area under receiver operating characteristic curve (AUROC) of 0.88 (95% confidence interval (CI), 0.86-0.94) for detecting patients with CRC (Supplementary Fig. 3a), and the average AUROC of fungal classification classifier was 0.76 (95% CI, 0.67-0.82; Supplementary Fig. 3b). However, the predictive capability of archaeal and viral species was limited, with no species from these kingdoms satisfying the criteria for effective features.

**Fig. 4:**
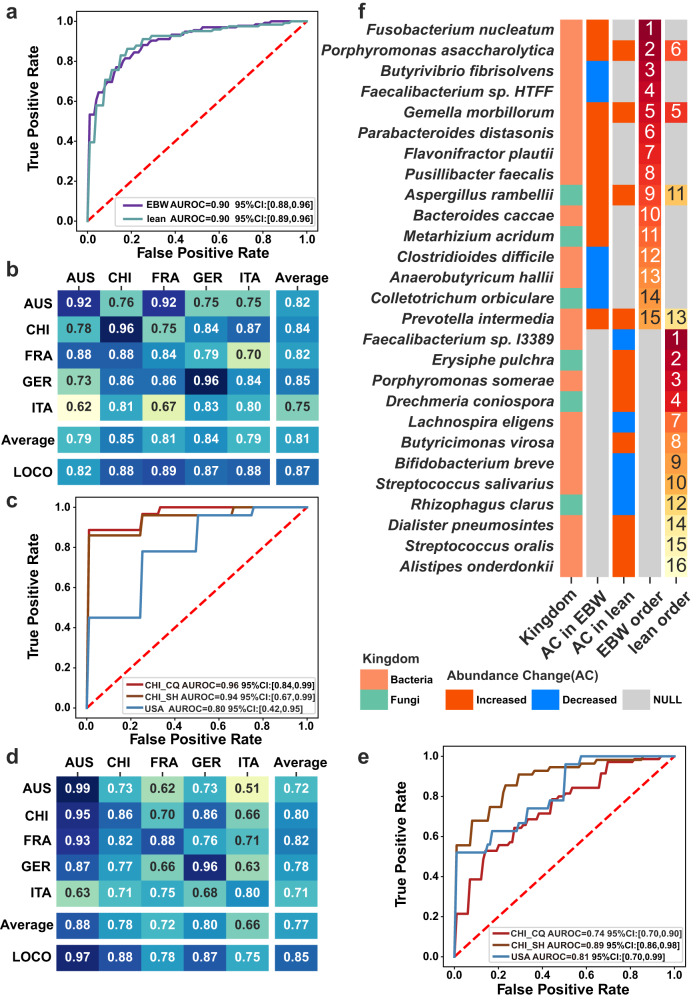
Diagnostic models constructed with multi-kingdom microbial signatures for EBW-CRC and lean-CRC: performance, validation, and feature importance. **a** Receiver operating characteristic (ROC) curves of five-fold-cross-validations on diagnostic models for EBW-CRC and lean-CRC with the discovery cohorts. **b**, **d** The AUROC matrix of internal cross-validations including cohort-to-cohort validation and LOCO validation on microbial signatures for distinguishing EBW-CRC from EBW-CTR **b** and distinguishing lean-CRC from lean-CTR **d**. The values in the cohort matrix refer to AUROC obtained by training the model on the cohort of corresponding row and applying it to the cohort of corresponding column. The values in LOCO row refers to AUROC obtained by training model on all but the cohort of the corresponding column and applying it to the cohort of corresponding column. **c**, **e** The AUROCs of five-fold-cross-validation on identified signatures for EBW-CRC **c** and lean-CRC **e** patients with three external validation cohorts. **f** Feature importance of the microbial markers for EBW and lean. The first column plots the kingdom information of the microbial markers, followed by two columns of color-coded information on the alteration of the abundances of signatures in EBW-CRC and lean-CRC, respectively. The last two columns list the rank of importance for EBW-CRC and lean-CRC signatures, respectively.

Subsequently, we combined multi-kingdom features in expect for superior distinguishing capability. Owing to the relatively low distinguishing capability, the viral and archaeal species were removed during the differential feature selection. Therefore, the optimal multi-kingdom classifier achieving an average AUROC of 0.90 (95% CI, 0.88–0.96) was constructed with bacterial and fungal species (Fig. 4a), slightly superior to the bacterial classifier. Nevertheless, the signature importance analysis underlined three fungal species, *A. rambellii*, *M. acridum* and *C. orbiculare*, that ranked 9^th^, 11^th^and 14^th^ for diagnosing EBW-CRC. Note that most of bacterial signatures, such as *F. nucleatum*, *Porphyromonas ascaccharolytica*, *G. morbillorum* and *C. difficile* have been reported as important biomarkers for CRC^24,28,42^.

In addition, we assessed the detecting capability of functional models based on differential KO genes. The best performing functional model was constructed with 33 KO signatures, achieving an average AUROC of 0.86 (95% CI, 0.85-0.95; Supplementary Fig. 3c), which is inferior to the performance of the optimal multi-kingdom species model.

#### Evaluation of the robustness and disease specificity of EBW-CRC signatures

To evaluate the generalization and robustness of the best multi-kingdom signatures for EBW-CRC, we performed cohort-to-cohort and leave-one-cohort-out (LOCO) validation^27^. The AUROC ranged from 0.62 to 0.96 with an average of 0.81 in cohort-to-cohort validation, and further improved in LOCO validation, ranging from 0.82 to 0.89 with an average of 0.87 (Fig. 4b). Further, the robustness of signatures was validated by three independent cohorts, with AUROC of 0.96 (95% CI, 0.84-0.99), 0.94 (95% CI, 0.67–0.99) and 0.80 (95% CI, 0.42–0.95) for CHI_CQ, CHI_SH and USA cohort, respectively (Fig. 4c).

These years have seen the distinctive capability of microbial signatures for varieties of diseases^27,28,43,44^, thus, it is indispensable to further appraise the disease specificity of the EBW-CRC signatures with cohorts affected by other microbiome related diseases and even lean-CRC. To this end, we tested the signatures’ disease specificity with cohorts of inflammatory bowel disease (IBD), liver cirrhosis (LC), and lean-CRC (Details were described in the method). The AUROC decreased by 1.37%, 0.72%, and 5.27% when adding the diseased samples from the IBD cohort to the control group of each external EBW-CRC validation cohort compared to adding healthy controls samples of IBD cohort (Supplementary Fig. 4). Such variations of AUROC values were slight without significance, considering the baseline of altered AUROC when adding EBW-CRC samples or control samples to the external validation cohorts, which dramatically decreased by 11.65%, 8.39%, and 19.69%, respectively (Supplementary Fig. 4). Similar results were observed when tested with the LC cohort (decreased by 1.68%, 2.57%, and 4.99%, respectively, Supplementary Fig. 4) and lean-CRC individuals with AUROC decreasing by 0.81% and 0.37% and increasing by 2.20% (Supplementary Fig. 4). Taken together, these results demonstrated the disease specificity of the identified microbial signatures for EBW-CRC.

#### Identification and validation of lean-CRC associated microbial signatures

Similarly, we identified microbial signatures for lean-CRC. Among the single-kingdom classification models, the bacterial model (AUROC = 0.87; 95% CI, 0.85–0.94; Supplementary Fig. 5a) outperformed the fungal model (AUROC = 0.74; 95% CI, 0.67–0.84, Supplementary Fig. 5b) in classifying lean-CRC. For the viral kingdom, only one species from the kingdom passed the feature selection process, and displayed poor predictive power for lean-CRC with the same AUROC of 0.56 (95% CI, 0.45–0.62; Supplementary Fig. 5c).

We then tested the efficacy of the classifier constructed with the combination of multi-kingdom signatures (Supplementary Data 18). The classifier based on bacteria-fungi signatures achieved the highest AUROC of 0.90 (95% CI, 0.89–0.96; Fig. 4a). Included in this classifier were 12 bacterial- and 4 fungal- species. This classifier was validated for generalization in cohort-to-cohort validation achieving an average AUROC of 0.77 and in LOCO validation achieving an average AUROC of 0.85 (Fig. 4d). Furthermore, the robustness of the classifier was evaluated with three additional cohorts achieving average AUROCs of 0.74 (95% CI, 0.70–0.90), 0.89 (95% CI, 0.86–0.98) and 0.81(95% CI, 0.70–0.99) for cohorts CHI_CQ, CHI_SH and USA, respectively (Fig. 4e. Consistent with EBW-CRC, the performance of functional KO gene classifier (AUROC = 0.83; 95% CI, 0.82–0.92; Supplementary Fig. 5d) was not comparable to that of the optimal multi-kingdom classifier in distinguishing lean-CRC.

Next, similar to the signatures for EBW-CRC, the disease specificity of signatures for lean-CRC was validated. The average change of AUROC is 2.06% in IBD cohort, -1.63% in LC cohort and -2.25% in EBW-CRC cohort, which is slight variation since the baseline values of altered AUROC was around -10.20% on average (Supplementary Fig. 6). These data demonstrated the disease specificity of the identified microbial signatures for lean-CRC.

#### Characteristics of the signatures for EBW-CRC and lean-CRC

We investigated the signatures distribution in both EBW-CRC and lean-CRC models, and found substantial variations between the two classifiers, with only four common signatures including *P. asaccharolytica, G. morbillorum, A. rambellii* and *P. intermedia* (Fig. 4f). All these signatures displayed increased abundance in both EBW-CRC and lean-CRC. Among these, *P. asaccharolytica* ranked 2/15 and 6/16 among the signatures for EBW-CRC and lean-CRC, respectively. *G. morbillorum* has been reported as a signature for CRC patients in previous studies^25,26,28,45^ and shown similar capability for both EBW-CRC (ranking as 5^th^) and lean-CRC (ranking as 5^th^), respectively (Fig. 4f). In addition, the other top bacterial signatures in excess body weight population, specifically, included the widely-reported *F. nucleatum* and *C. difficile*, as well as short-chain fatty acid-producing bacteria *A. hallii* and butyrate-producing bacteria *B. fibrisolvens*. Meanwhile, the most important signatures specific for lean-CRC included *F. sp. I3389*, *E. pulchra and Porphyromonas somerae*. These results highlight the importance of identifying EBW- and lean-specific microbial signatures.

#### Predictable capability of signatures for early-onset CRC

Furthermore, considering the steadily rising incidence of early-onset CRC (diagnoses before the age of 50), we explored the potential of our signatures for detecting early-onset CRC using available samples in the discovery cohort. The five-fold cross-validation model achieved an average AUROC of 0.93 for both EBW-CRC (Supplementary Fig. 7a) and lean-CRC (Supplementary Fig. 7b).

### Taxonomic and functional association characteristics

**Fig. 5:**
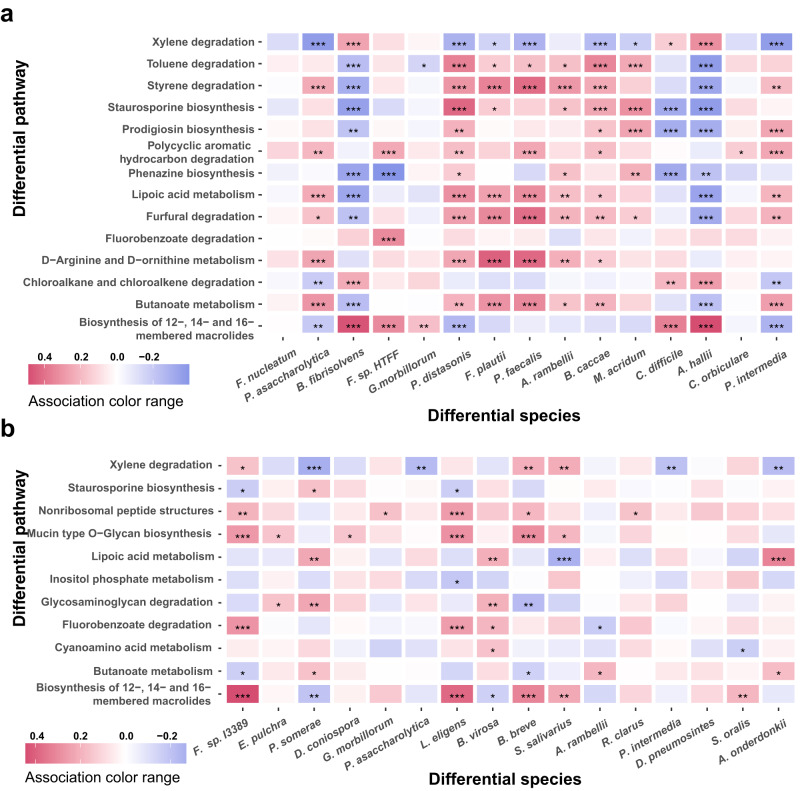
The associations between differential species and differential pathways. Heatmaps show the correlation coefficients of association between differential species and pathways in EBW-CRC **a** and lean-CRC **b**, respectively. Color red represents for positive association and blue for negative association. *P* values, FDR-corrected *P* values, and correlation coefficients were computed via Hierarchical All-against-All association testing. Asterisks indicate statistical significance (*, FDR-corrected *P* ≤ 0.05; **, FDR-corrected *P* ≤ 0.01; ***, FDR-corrected *P* ≤ 0.001).

To understand the functional relevance of the microbial alterations for EBW and lean patients with CRC, we analyzed the associations between the differential microbiota and the differential pathways within each group. Numerous associations were identified, displaying distinct association patterns between the EBW and the lean populations (Supplementary Data 19 and Supplementary Data 20). For excess body weight patients, several differential pathways, such as lipoic acid metabolism, furfural degradation, D-Arginine and D-ornithine metabolism and butanoate metabolism, were positively associated with majority of signatures (Fig. 5a). Furthermore, *P. intermedia* were negatively associated with a few differential pathways including xylene degradation in lean group but were positively associated with several differential pathways including butanoate metabolism, furfural degradation, lipoic acid metabolism and prodigiosin biosynthesis in EBW group (Fig. 5). And many signatures for lean patients were associated with differential pathways including biosynthesis of 12-, 14- and 16-membered macrolides, mucin type O-Glycan biosynthesis, glycosaminoglycan degradation, xylene degradation, butanoate metabolism and lipoic acid metabolism. And some important signatures, *P. somerae*, *Lachnospira eligens*, *F. sp. I3389*, were associated with many important differential pathways in lean patients (Fig. 5b).

## Discussion

Considering the increased risk of CRC associated with excessive body weight^8^, we characterized the gut microbiota of the overweight-obesity and lean patients with CRC, using metagenomic sequencing data generated from multiple cohorts of various geographical and cultural backgrounds. Distinct microbial taxonomic and functional alterations in EBW and lean patients with CRC were observed. Notably, specific optimal panels of multi-kingdom microbial signatures were identified for EBW-CRC and lean-CRC with AUROC values of 0.90 and 0.90, respectively. The robustness and disease specificity of these signatures were validated with three independent cohorts. Given the challenges in the detection of CRC with excess body weight population^21,22^, and the non-invasive nature of microbial signatures, the highly sensitive and specific microbial signatures we identified are promising to serve as adjunctive tools for CRC diagnosis, especially for EBW-CRC.

CRC is associated with altered microbial composition^9,36,46^. On top of this, increased body weight and obesity, known for considerable impact on the gut microbiota, are associated with higher risks of CRC^8,47^. Thus, it is of particular importance to investigate the microbial features of excess body weight patients with CRC, in comparison with that in lean patients with CRC. Abundant differences in the microbial composition between EBW-CRC and lean-CRC were observed, which was reflected by the EBW-CRC specific increase in alpha diversity. We made several outstanding observations among these differences. Firstly, while enriched abundances of *P. micra, P. asaccharolytica, B. fragilis, G. morbillorum*, *A. rambellii*, and *F. nucleatum* were common for both EBW-CRC and lean-CRC, in consistence with previous findings with general CRC populations^25,26,28^, our results demonstrated EBW-CRC specific abundance changes in *C. comes*, *P. distasonis*, *C. difficile* and *F. plautii*. Among these, *C. difficile* could drive tumorigenesis of CRC by secreting toxin TcdB^42^. It is interesting to note that previous study has reported an association between the abundance of *C. difficile* and obesity^48^, suggesting that *C. difficile* may mediate a link between obesity and elevated CRC incidence. Secondly, we observed that *F. prausnitzii*, previously reported to be depleted in CRC^24,26,49^, was specifically decreased in lean-CRC. It has been reported that *F. prausnitzii* played a crucial role in suppressing CRC via converting 7-dehydrocholesterol into vitamin D and subsequently activating the host VDR^41^. Our results suggest that this mechanism may only apply to the lean population of CRC. Thirdly, we noticed that, *P. distasonis*, considered a protective species attenuating colon tumor formation via blocking toll-like receptor 4 signaling pathway and Akt activation in HFD-induced CRC mice, and exhibiting decreased abundance in the CRC mice^12^, was specifically elevated in EBW-CRC but not in lean-CRC, indicating a possible difference between the gut microbiota of mice and human.

Importantly, large amount of differences were observed in the inter-kingdom interactions between the EBW-CRC and the lean-CRC. Notably, the association of *A. rambellii* and *F. nucleatum* may be one factor contributing to CRC pathogenesis^35^. We found that this association was only observed in EBW-CRC but not in lean-CRC, suggesting a potential mechanism that may contribute to the differential pathogenesis between EBW-CRC and lean-CRC.

Out of these differential multi-kingdom species, we identified optimal panels of signatures for distinguishing EBW-CRC and lean-CRC from controls, respectively. These two panels were robustly validated in cohort-to-cohort and LOCO validations with three independent cohorts, and displayed satisfactory disease specificity with cohorts of other microbiota-related diseases. It is noteworthy that, some previously reported CRC biomarkers, such as *F. nucleatum*, *C. difficile*, as well as short-chain fatty acid-producing bacteria *A. hallii* and butyrate-producing bacteria *B. fibrisolvens*, were identified as EBW-CRC specific signatures in our study, while *L. eligens*, *B. breve* and *E. pulchra*, were identified as specific to lean-CRC^28,35,36,42,50^. Particularly, *L. eligens*, a butanoate-producing probiotic, was capable of suppressing inflammation and preventing colitis and CRC^51^. Similarly, butyrate producer *B. fibrisolvens*, when paired with a high-fiber diet, also has demonstrated anti-CRC effects^52^. In addition, we identified additional lean-CRC specific signatures including *D. pneumosintes*, and *S. oralis*. The oral pathogen *D.pneumosintes* has been reported to be increased in advanced CRC^53,54^, while *S. oralis* is an oral peroxigenic bacteria. Enriched abundance of these two pathogens may contribute to cancer development. On the other hand, it is noteworthy that *B. breve*, an anti-tumor species^50^, was a lean-CRC specific signature exhibiting decreased abundance in lean-CRC. Besides these specific microbial signatures, we also identified common microbial signature for both EBW-CRC and lean-CRC, including *P*. *asaccharolytica, P. intermedia, G. morbillorum*, and *A. rambellii*, whose diagnostic values for CRC have been highlighted previously^27,28,55^. With stratified patient populations, we demonstrated that these species are reliable universal markers for both EBW- and lean-CRC.

The functional analysis strengthens the discrepancy in gut microbiome between EBW- and lean-CRC. As with the compositional analysis, unique microbial functions were identified in EBW-CRC and lean-CRC. On one side, increased metabolic potentials of D-Arginine and D-ornithine metabolism was specifically elevated in EBW-CRC. While previous studies suggested that these functional alterations are common features for CRC in general^28^, our closer examination pinpointed this pathway to be specific for EBW-CRC. On the other hand, pathways, inositol phosphate metabolism, synthesis, and degradation of ketone bodies were specifically enriched in lean patients with CRC. In addition, while our data consistently showed an increased abundance of butanoate metabolism in both EBW-CRC and lean-CRC compared to their respective controls, the exact mechanism and potential causation, given the discrepant effects of butyrate on tumourigenesis^9,56–58^, requires further exploration.

In conclusion, this comprehensive study unearthed unique characteristics of microbial compositions and functions in excess body weight and lean patients with CRC. To address the limitation of the lack of prospective cohorts, we conducted multidimensional validations to confirm the robustness, universality, and disease specificity of the identified microbial signatures. However, due to the unavailability of data related to dietary and lifestyle, as well as variable criteria for excess-weight in different regions, further investigations should take these aspects into account. Despite these limitations, this study has successfully identified specific multi-kingdom microbial signatures for both excess body weight and lean CRC individuals, underscoring their potential as accurate, non-invasive adjunctive tools for CRC screening, particularly in excess-weight populations. Additionally, there is an urgent need for relevant authorities and organizations to implement strategies to enhance public engagement in CRC screening.

## Materials and methods

### In-house data generation and public data collection

We included one in-house fecal shotgun metagenomic data in the study. CHI_SH cohort, collected in Shanghai, China, consisting 80 CRC patients and 86 healthy controls with similar ages. Fecal sampling was conducted when patients were initially diagnosed with no reception of any treatment. Written informed consent were obtained from all subjects before biospecimen collection. This metagenomic data was described and published in our previous study^28^.

We also collected publicly available fecal shotgun metagenomic data of human CRC patients and healthy controls. Raw sequencing data of seven cohorts from five countries were downloaded from the Sequence Read Archive using the following identifiers: ERP008729 for Feng et al.^46^, PRJEB10878 for Yu et al.^36^, ERP005534 for Zeller, G. et al.^59^, PRJEB27928 for Wirbel et al.^25^, SRP136711 for Thomas et al.^26^, PRJNA429097 for Yang et al.^60^, and PRJEB12449 for Vogtmann et al.^61^. We manually curated metadata from relevant original publications. Individuals were stratified according to their BMI. The excess body weight group include subjects who are overweight (BMI$\ge$25 kg/m^2^) or obese (BMI$\ge$30 kg/m^2^), and lean groups includes subjects with normal body weight (BMI < 25 kg/m^2^) according to the criteria of World Health Organization^16^.

Since this study aims to pinpoint robust and universal EBW-specific microbial signatures across global cohorts, based on the available data, we selected samples from Austria, China, France, Germany, and Italy, diverse in geography and culture, as our discovery/training datasets. All other samples served as validation sets. Notably, from the three Chinese datasets, samples from HongKong (PRJEB10878) were randomly chosen for discovery. The USA dataset, with specimens freeze-archived for over 25 years before metagenomic sequencing^25,61^, was specifically used for validation to mitigate potential biases in microbial markers identification. Besides, two cohorts of samples with gut microbiota related diseases including liver cirrhosis (LC) and Inflammatory bowel disease (IBD) under accession number PRJEB6337^62^ and PRJNA398089^63^, respectively, were included to evaluate the specificity of signatures for EBW- and lean- CRC patients, respectively.

### Metagenome data preprocessing and annotation

Firstly, we used KneadData v.0.6 to remove low-quality reads and contaminant reads which included host-associated and laboratory-associated sequences by bowtie2 v.2.3.5. Thereafter, Kraken2 was utilized to perform metagenomic taxonomy classification against our customized reference database. The customized database comprises 32,875 bacterial, 489 archaeal, 11,694 viral reference genomes from the National Center for Biotechnology Information Refseq database (accessed on August 2022), and 1,256 fungal reference genomes from the National Center for Biotechnology Information Refseq database, FungiDB (http://fungidb.org) and Ensembl (http://fungi.ensembl.org) (accessed on August 2022). It was built using the Jellyfish program by counting distinct 31-mer in the reference libraries, with each k-mer in a read mapped to the lowest common ancestor of all reference genomes with exact k-mer matches. And taxonomic abundance was accurately counted by Bracken v.2.5.0. For taxonomic profiles, the absolute abundances obtained above were transformed into relative abundances. Next, function profiles were generated^28^, mainly including reads assembling into contigs via Megahit v.1.2.9, genes prediction by Prodigal v.2.6.3, non-redundant microbial gene set construction by CD-HIT. EggNOG mapper v.2.0.1 was used to annotate genes and gene abundance was estimated with CoverM v.4.0 by calculating the coverage of genes in the original contigs. The relative abundances of KEGG KO groups or pathways were calculated by summing the relative abundances of corresponding genes based on annotation results.

### Identification of differential microbial signatures across cohorts

Considering that the heterogeneity among cohorts exerts considerable impact on microbial profiles, we used *xMarkerFinder*^64^, an integrated platform to conduct the following analyses: differential signature identification, model construction, model validation, and signatures interpretation. The detailed procedures are described below.

#### Identification of microbial differential signatures

Due to sparsity of microbial abundance matrix, microbial compositional profiles were filtered with the following criteria: (1) Microbial species that did not exceed a maximum average relative abundance of 0.001% in at least two of the studies were excluded; (2) Microbial species with mean relative abundance below 0.01% were excluded; (3) Microbial species with prevalence below 20% were excluded. Next, differential microbes between CRC and CTR were identified by R package MMUPHin v.1.4.2 with *P* < 0.05 and FDR-corrected *P* < 0.1.

Similar preprocessing was performed against microbial KO genes profile and genes with *P* < 0.05 and FDR-corrected *P* < 0.1 identified by MMUPHin v.1.4.2.

Additionally, batch effect was eliminated during differential analysis by designating the cohort as main batch effect according to the PERMANOVA results, with demographic characteristics, including gender, age, and BMI, selected as covariates if *P* < 0.05 in the PERMANOVA analysis.

#### Candidate signature selection for classifier

Based on differential signatures described above, an integrated signature selection analysis in *xMarkerFinder*^64^ were performed to determine potential signature to distinguish overweight-obesity or lean CRC. This process consists of three-step feature selection procedure in turn, namely effective feature selection, collinear feature exclusion and recursive feature elimination. AUROC threshold of effective feature selection was the default setting of *xMarkerFinder*. Features with a high correlation coefficient (above 0.8) were considered collinear. The optimal features in recursive feature elimination were considered as candidate signatures.

#### Classifier construction and evaluation

Candidate signatures in species and function levels were utilized to construct random forest model. Hyperparameters, such as the number of estimator trees, the maximum depth of the trees, the numbers of features per tree, and the maximum samples was tuned to optimize the classifier via bayesian-optimization v.1.2.0 package in Python. The best classifier was constructed by the optimal signature combination and the optimal hyperparameters.

We conducted receiver operating characteristic (ROC) analysis, and calculated AUROCs to evaluate the performance of our classification models using the Python package sklearn. The average AUROC, a widely accepted measure of central tendency, was reported for each model. The 95% confidence interval of AUROC was estimated by bootstrapping.

We further evaluated the generalization of signatures through cohort-to-cohort validation and LOCO validation^27,28^. For cohort-to-cohort validation, the diagnostic model was trained on one cohort using signatures and then validated in the other cohort. For LOCO validation, one cohort was sequentially excluded for validation, each time the remaining cohorts were used to construct diagnostic model based on signatures. In addition, the robustness of signatures was validated with three independent cohorts.

#### Disease specificity assessment of signatures

To assess the disease specificity of the signatures, two non-CRC disease cohorts, LC and IBD, were collected. Furthermore, the disease specificity of EBW-CRC signatures on lean-CRC individuals from ERP005534 was also evaluated using the same method, and vice versa. The approach for disease specificity validation^26,30^ is akin to the “Difference in difference” statistical technique that could compare AUROC values within the same dataset, ensuring equivalent batch effects when comparing non-CRC and original CRC datasets. Briefly, 15 randomly selected diseased or healthy subjects from non-CRC dataset, were respectively added into the control group of the external CRC validation cohort, and AUROC values were calculated with the original models. For disease specific signatures, it is expected that adding diseased or control samples from cohorts of other disease will not cause significant change in performance of original model constructed in the external CRC validation cohort, since these signatures have no distinguishing capability for non-CRC samples. For comparison, we further set a baseline of the AUROC alterations by adding the CRC samples or relevant controls samples of ERP005534 into the control group of the external CRC validation cohort. The procedure was repeated for ten times. Notably, considering there were only seven excess body weight healthy subjects available in LC cohort, five cases and five controls were randomly sampled for this procedure.

#### Co-abundance analysis

FastSpar v.1.0.0 was used to analyze co-abundance associations among species. Absolute abundances of differential microbial species were used to estimate correlation coefficients and construct co-abundance network based on SparCC algorithm, which is suitable for sparsely populated compositional data to mine correlations among microbes. The co-abundance relationship with FDR-corrected *P* < 0.05 were defined as significant associations. Among these, strong co-abundance correlations were defined using the cut-off of the absolute value of correlation coefficient above 0.3. Significant association networks and strong correlation networks were constructed in different disease status including EBW-CRC, EBW-CTR, lean-CRC, and lean-CTR, respectively. Networks were visualized with Gephi v.0.10.1.

### Characterization of microbe-pathway interaction

To investigate the interactions between microbial species and function, we performed Hierarchical All-against-All association testing, a computational method to integrate multi-omics data based on Spearman correlation. The associations with FDR-corrected *P* < 0.05 were considered potential interactions between microbial species and pathways for further interaction analysis.

### Statistics and reproducibility

No statistical method was used to predetermine sample size, since this is an integrated analysis based on public metagenome data with enough samples. No data were excluded from the analyses. The experiments were not randomized because statistical analyses depended on information about cancer status. Data collection and analysis were not performed blind to the conditions of the experiments. Alpha diversity of all kingdoms was calculated in each sample using Shannon Index metrics with R package ‘vegan’. And the significance of alpha diversity was assessed by Wilcoxon rank sum test. Beta diversity was evaluated using Bray-Curtis distance. The differential significance of beta diversity among 4 groups were assessed by permutational multivariate analysis of variate (PERMANOVA) with 999 permutations. PERMANOVA was also utilized to perform confounder analysis, which quantified the impact of the metadata variables on microbial profiles using R v4.0.5 “vegan” v2.5.7 package. We treated variable with predominant impact as major batch effect according to *P* value and ${R}^{2}$ of each metadata variable. Remained variables with *P* < 0.05 were set as covariates. False discovery rate control for multiple testing was made using the Benjamini-Hochberg adjustment. All analyses were implemented with R v.4.0.5 and Python v.3.8.13, and visualized with R v.4.0.5, Python v.3.8.13 and Gephi v.0.10.1.

### Reporting summary

Further information on research design is available in the Nature Portfolio Reporting Summary linked to this article.

### Supplementary information

Supplementary Information

Description of Additional Supplementary Files

Supplementary Data 1-20

Supplementary Data 21

Reporting Summary

## Data Availability

All supporting data has been provided in Supplementary Data 1-20 and source data in Supplementary Data 21. All raw data in our work are publicly available. In-house metagenomics data of CHI_SH cohort were deposited in the National Omics Data Encyclopedia (NODE) (https://www.biosino.org/node/) with accession code OEP001340. Other publicly available metagenomics data can be found at the Sequence Read Archive (https://www.ncbi.nlm.nih.gov/sra) and European Nucleotide Archive (https://www.ebi.ac.uk/ena/) under accession numbers ERP008729, PRJEB10878, ERP005534, PRJEB27928, SRP136711, PRJNA429097, PRJEB12449, PRJEB6337 and PRJNA398089.

## Code availability

The primary codes and scripts are available at https://github.com/tjcadd2020/xMarkerFinder.
