# Supplementary material for: Multi-kingdom microbial signatures in excess body weight colorectal cancer based on global metagenomic analysis
Source: Commun Biol. 2024 Jan 5;7:24. doi: 10.1038/s42003-023-05714-0 (PMC10770074; doi:10.1038/s42003-023-05714-0)
Supplement: Supplementary file 1 — Supplementary Information [file 42003_2023_5714_MOESM1_ESM.pdf]

Supplementary Figures

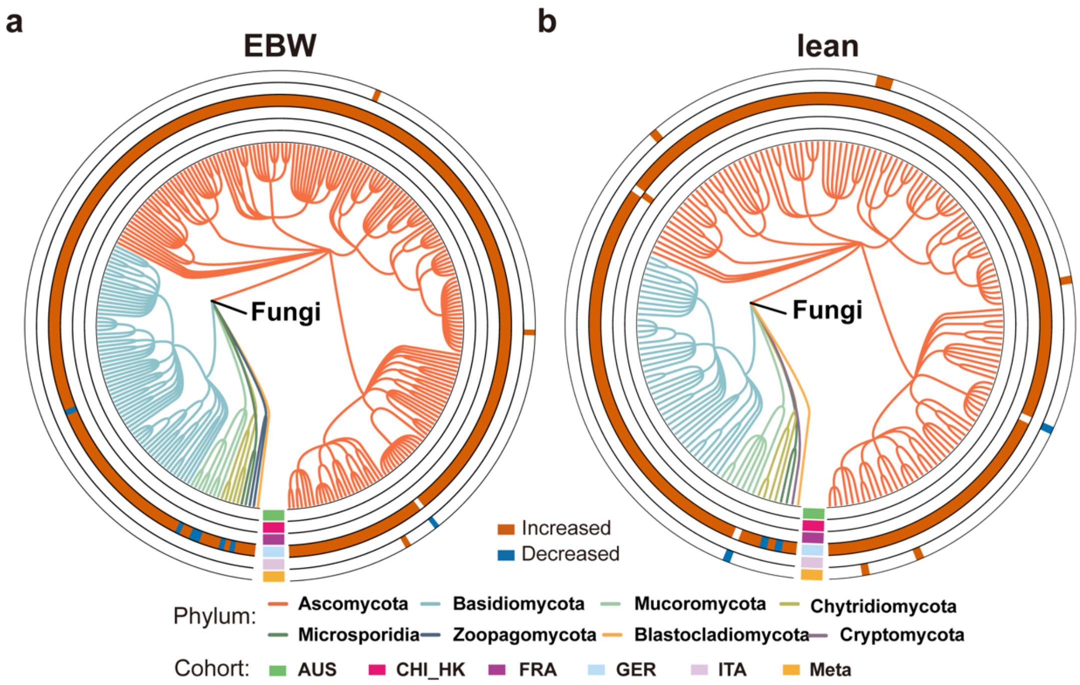

Supplementary Fig. 1: Differential fungal species. Phylogenetic tree of differential fungal species in EBW-CRC (a, 271 species) and lean-CRC (b, 192 species). The outer circles display differential species (FDR-corrected *P*<0.1) in each cohort and in the meta-analysis. Red and blue indicate increased or decreased abundance.

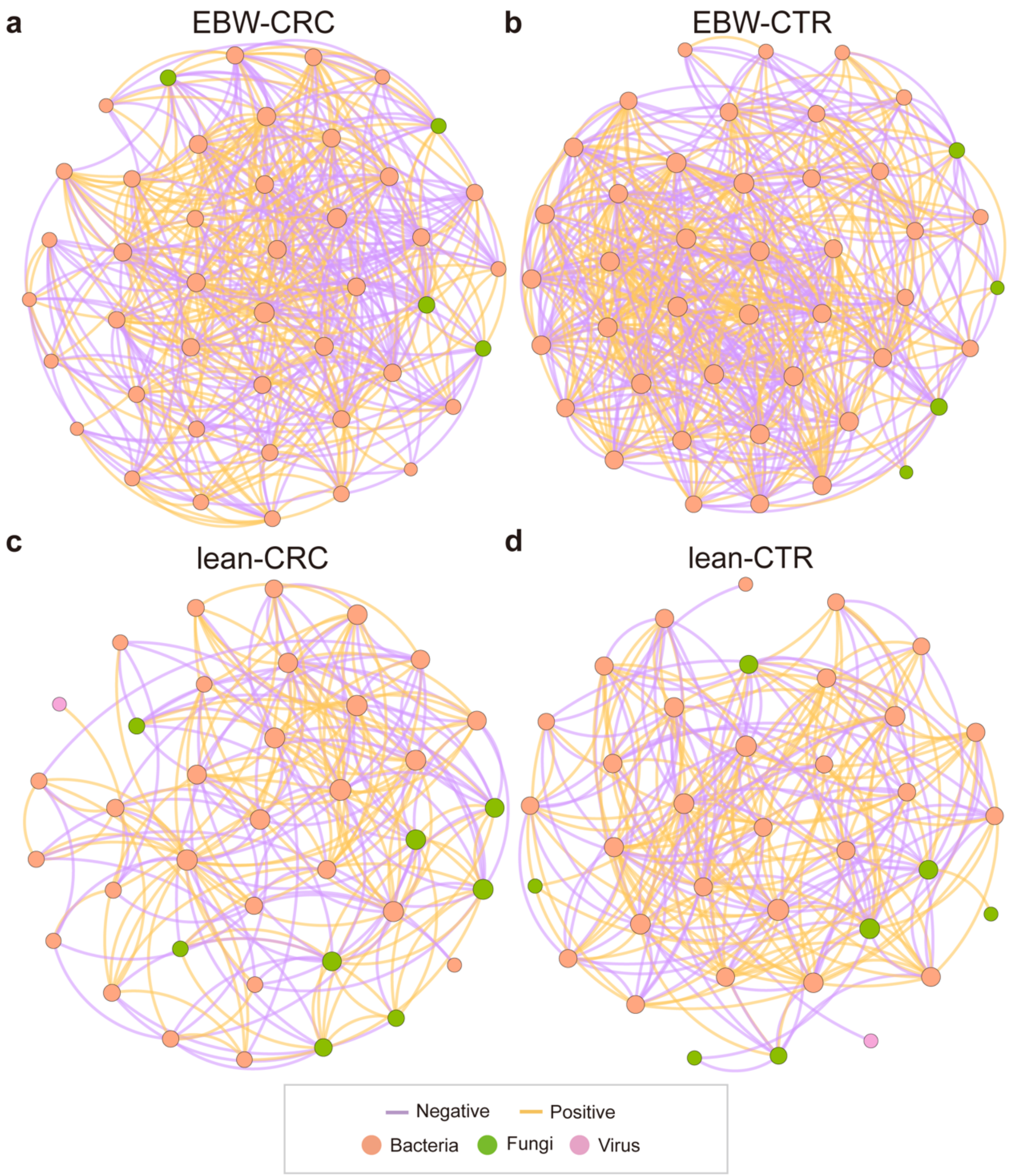

Supplementary Fig. 2: Microbial co-abundance networks in EBW-CRC(a), EBW-CTR(b), lean-CRC(c), and lean-CTR(d). Each co-abundance network consists of significant associations (FDR-corrected $P<0.05$) among differential species. Node colors indicate different kingdoms: bacteria(orange), fungi(green), virus(pink). Line colors indicate positive (orange) or negative (purple) correlations.

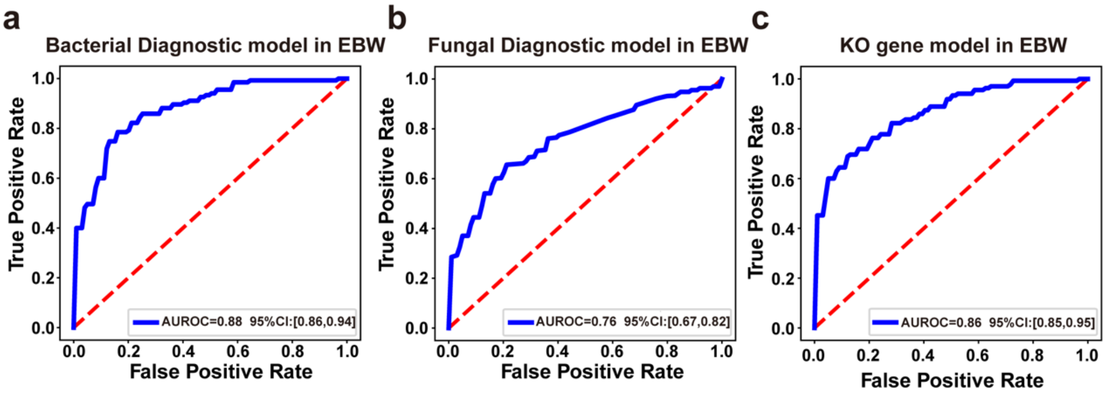

Supplementary Fig. 3: The performance of single kingdom and functional diagnostic models for EBW-CRC patients. (a, b) The performance of five-fold-cross-validation of bacterial (a) and fungal (b) diagnostic models for EBW-CRC. (c) The performance of diagnostic models based on KO gene markers for EBW-CRC.

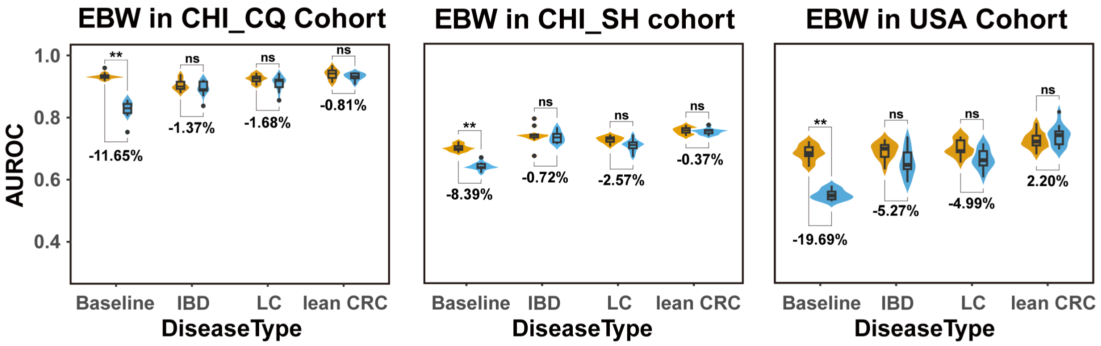

Supplementary Fig. 4: Disease specificity of the microbial signatures based on the optimal panel for EBW-CRC. Validation of the disease specificity of signatures for EBW-CRC against other microbiome related diseases (LC, IBD and lean-CRC) with three external validation cohorts of EBW. The blue bars indicate performance of models when adding diseased samples (baseline: excess body weight colorectal cancer, IBD: inflammatory bowel diseases, LC: liver cirrhosis, or lean-CRC: lean colorectal cancer) into the control group of each validation cohort. The orange bars indicate performance of models when adding corresponding healthy controls (from the cohorts of EBW-CRC, IBD, LC, lean-CRC) into the control group of each validation cohort. Wilcoxon rank sum test was conducted to evaluate significance in each comparison of AUROCs. And false discovery rate control was made using the Bonferroni adjustment. Asterisks indicate statistical significance (*, FDR-corrected $P \leq 0.05$; **, FDR-corrected $P \leq 0.01$; ***, FDR-corrected $P \leq 0.001$). Percentage represents the change of AUROC when adding diseased samples compared to adding controls.

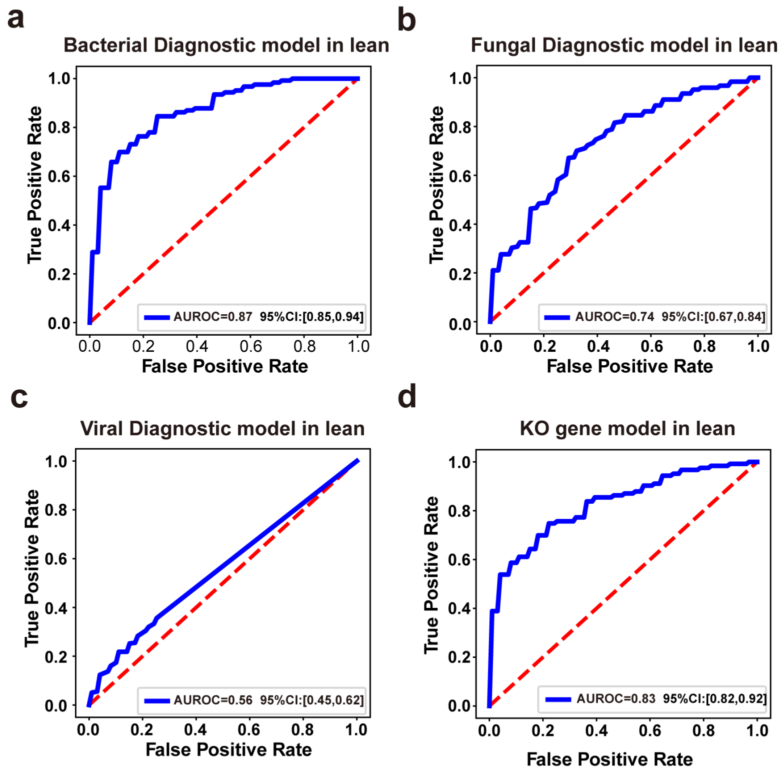

Supplementary Fig. 5: The performance of single kingdom and functional diagnostic models for lean-CRC patients. (a, b, c) The performance of five-fold-cross-validation of bacterial (a), fungal (b) and archaeal(c) diagnostic models for EBW-CRC. (d) The performance of diagnostic models based on KO gene markers for lean-CRC.

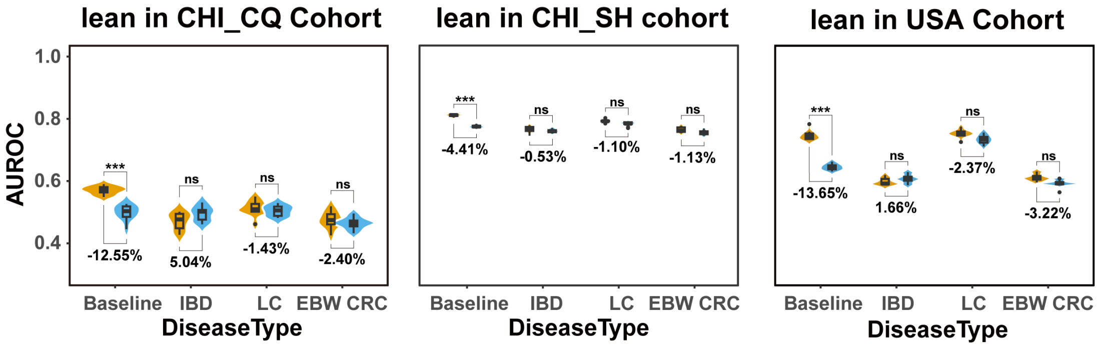

Supplementary Fig. 6: Disease specificity of the microbial signatures based on the optimal panel for lean-CRC. Validation of the disease specificity of signatures for lean-CRC against other microbiome related diseases (LC, IBD and EBW-CRC) with three external validation cohorts of lean. The blue bars indicate performance of models when adding diseased samples (baseline: lean colorectal cancer, IBD: inflammatory bowel diseases, LC: liver cirrhosis, or EBW-CRC: excess body weight colorectal cancer) into the control group of each validation cohort. The orange bars indicate performance of models when adding corresponding healthy controls (from the cohorts of lean-CRC, IBD, LC, EBW-CRC) into the control group of each validation cohort. Wilcoxon rank sum test was conducted to evaluate significance in each comparison of AUROCs. And false discovery rate control was made using the Bonferroni adjustment. Asterisks indicate statistical significance (*, FDR-corrected $P \leq 0.05$; **, FDR-corrected $P \leq 0.01$; ***, FDR-corrected $P \leq 0.001$). Percentage represents the change of AUROC when adding diseased samples compared to adding controls.

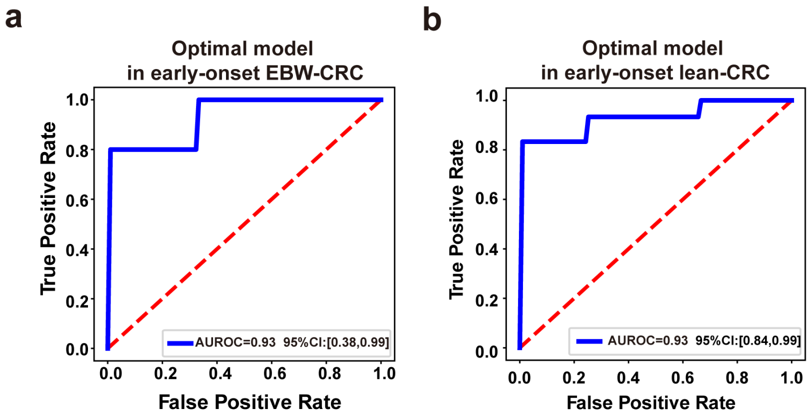

Supplementary Fig. 7: The performance of microbial signatures in early-onset individuals. (a) The performance of diagnostic models in early-onset CRC individuals based on optimal multi-kingdom biomarkers for EBW-CRC. (b) The performance of diagnostic models in early-onset CRC individuals based on optimal multi-kingdom biomarkers for lean-CRC.
