# Supplementary material for: Multi-kingdom microbial signatures in excess body weight colorectal cancer based on global metagenomic analysis
Source: Commun Biol. 2024 Jan 5;7:24. doi: 10.1038/s42003-023-05714-0 (PMC10770074; doi:10.1038/s42003-023-05714-0)
Supplement: Supplementary file 2 — Description of Additional Supplementary Files [file 42003_2023_5714_MOESM2_ESM.pdf]

Description of Additional Supplementary Files

File name: Supplementary Data 1
Description: Characteristics of fecal metagenomics samples included in the study

File name: Supplementary Data 2
Description: Differential microbial species between EBW-CRC and EBW-CTR

File name: Supplementary Data 3
Description: Differential microbial species between lean-CRC and lean-CTR

File name: Supplementary Data 4
Description: Common differential species between EBW-CRC and lean-CRC

File name: Supplementary Data 5
Description: Differential species specific for EBW-CRC

File name: Supplementary Data 6
Description: Differential species specific for lean-CRC

File name: Supplementary Data 7
Description: Co-abundance correlations in EBW-CRC

File name: Supplementary Data 8
Description: Co-abundance correlations in EBW-CTR

File name: Supplementary Data 9
Description: Co-abundance correlations in lean-CRC

File name: Supplementary Data 10
Description: Co-abundance correlations in lean-CTR

File name: Supplementary Data 11
Description: Differential KO genes between EBW-CRC and EBW-CTR

File name: Supplementary Data 12
Description: Differential KO genes between lean-CRC and lean-CTR

File name: Supplementary Data 13
Description: Common differential KO genes between EBW-CRC and lean-CRC

File name: Supplementary Data 14
Description: Differential KO genes specific for EBW-CRC

File name: Supplementary Data 15
Description: Differential KO genes specific for lean-CRC

File name: Supplementary Data 16
Description: Differential pathways between EBW-CRC and EBW-CTR

File name: Supplementary Data 17
Description: Differential pathways between lean-CRC and lean-CTR

File name: Supplementary Data 18
Description: Performances of the models constructed with different combinations of multi-kingdom signatures for lean-CRC

File name: Supplementary Data 19
Description: Associations between differential species and differential pathways in EBW-CRC

File name: Supplementary Data 20
Description: Associations between differential species and differential pathways in lean-CRC

File name: Supplementary Data 21
Description: Numerical source data for all graphs and charts in the manuscript
