# Supplementary material for: Multi-kingdom microbial signatures in excess body weight colorectal cancer based on global metagenomic analysis
Source: Commun Biol. 2024 Jan 5;7:24. doi: 10.1038/s42003-023-05714-0 (PMC10770074; doi:10.1038/s42003-023-05714-0)
Supplement: Supplementary file 5 — Reporting Summary [file 42003_2023_5714_MOESM5_ESM.pdf]

nature portfolio

Corresponding author(s): Ruixin Zhu, Lixin Zhu, Na Jiao

Last updated by author(s): Dec 2, 2023

Reporting Summary

Nature Portfolio wishes to improve the reproducibility of the work that we publish. This form provides structure for consistency and transparency in reporting. For further information on Nature Portfolio policies, see our Editorial Policies and the Editorial Policy Checklist.

Statistics

For all statistical analyses, confirm that the following items are present in the figure legend, table legend, main text, or Methods section.

n/a	Confirmed	
☐	☒	The exact sample size (*n*) for each experimental group/condition, given as a discrete number and unit of measurement
☐	☒	A statement on whether measurements were taken from distinct samples or whether the same sample was measured repeatedly
☐	☒	The statistical test(s) used AND whether they are one- or two-sided<br>*Only common tests should be described solely by name; describe more complex techniques in the Methods section.*
☐	☒	A description of all covariates tested
☐	☒	A description of any assumptions or corrections, such as tests of normality and adjustment for multiple comparisons
☐	☒	A full description of the statistical parameters including central tendency (e.g. means) or other basic estimates (e.g. regression coefficient) AND variation (e.g. standard deviation) or associated estimates of uncertainty (e.g. confidence intervals)
☐	☒	For null hypothesis testing, the test statistic (e.g. *F*, *t*, *r*) with confidence intervals, effect sizes, degrees of freedom and *P* value noted<br>*Give P values as exact values whenever suitable.*
☒	☐	For Bayesian analysis, information on the choice of priors and Markov chain Monte Carlo settings
☒	☐	For hierarchical and complex designs, identification of the appropriate level for tests and full reporting of outcomes
☐	☒	Estimates of effect sizes (e.g. Cohen's *d*, Pearson's *r*), indicating how they were calculated

Our web collection on statistics for biologists contains articles on many of the points above.

Software and code

Policy information about availability of computer code

Data collection	Data was downloaded manually from public databases by SRA toolkit (v.3.0.1).
Data analysis	KneadData (http://huttenhower.sph.harvard.edu/kneaddata, v.0.6), Bowtie2 (v.2.3.5), Kraken2, Braken (v.2.5.0)<br>Megahit (v.1.2.9), Prodigal (v.2.6.3), EggNOG mapper (v.2.0.1), CoverM (v.0.4.0, https://github.com/wwood/CoverM)<br>xMarkerFinder(v.1.0.14, https://github.com/tjcadd2020/xMarkerFinder), MMUPHin(v.1.4.2), bayesian-optimization (v.1.2.0)<br>vegan (v.2.5.7), sklearn (v.1.0.2), HAllA(v.0.8.17), FastSpar(v.1.0.0), R (v.4.0.5), Python (v.3.8.13), Gephi (v.0.10.1)

For manuscripts utilizing custom algorithms or software that are central to the research but not yet described in published literature, software must be made available to editors and reviewers. We strongly encourage code deposition in a community repository (e.g. GitHub). See the Nature Portfolio guidelines for submitting code & software for further information.

Data

Policy information about availability of data

All manuscripts must include a data availability statement. This statement should provide the following information, where applicable:

- Accession codes, unique identifiers, or web links for publicly available datasets
- A description of any restrictions on data availability
- For clinical datasets or third party data, please ensure that the statement adheres to our policy

All raw data in our work are publicly available. In-house metagenomics data of CHI_SH cohort were deposited in NODE (The National Omics Data Encyclopedia) with

accession code OEP001340. Other public metagenomics data were available at the Sequence Read Archive (SRA) (https://www.ncbi.nlm.nih.gov/sra) and European Nucleotide Archive (ENA) (https://www.ebi.ac.uk/ena/) under accession number ERP008729, PRJEB10878, ERP005534, PRJEB27928, SRP136711, PRJNA429097, and PRJEB12449.

Research involving human participants, their data, or biological material

Policy information about studies with human participants or human data. See also policy information about sex, gender (identity/presentation), and sexual orientation and race, ethnicity and racism.

Reporting on sex and gender	The gender of the participants is reported and considered as a covariate in the analysis of microbiome composition.
Reporting on race, ethnicity, or other socially relevant groupings	N/A
Population characteristics	All colorectal cancer and healthy control participants' characteristics including age, gender, BMI were recorded in various primary studies (see Supplementary Table 1). Considering the population heterogeneity, cohort was set as the batch and age, gender and BMI was treated as covariates.
Recruitment	This includes heterogeneous recruitment procedures performed in primary studies.
Ethics oversight	N/A

Note that full information on the approval of the study protocol must also be provided in the manuscript.

Field-specific reporting

Please select the one below that is the best fit for your research. If you are not sure, read the appropriate sections before making your selection.

☒ Life sciences ☐ Behavioural & social sciences ☐ Ecological, evolutionary & environmental sciences

For a reference copy of the document with all sections, see nature.com/documents/nr-reporting-summary-flat.pdf

Life sciences study design

All studies must disclose on these points even when the disclosure is negative.

Sample size	Our work combined 8 colorectal cancer cohort for a total of 981 samples. No sample size calculation was performed for this study, all publicly available data sets meeting a minimal set of inclusion criteria were included.
Data exclusions	Samples that lack BMI were removed from analysis.
Replication	We conducted multidimensional validations for our diagnostic biomarkers in three different cohorts of lean and excess body weight colorectal cancer patients and corresponding healthy controls.
Randomization	Not applicable for this observational case-control study.
Blinding	Blinding was not possible because statistical analyses depended on information about cancer status .

Reporting for specific materials, systems and methods

We require information from authors about some types of materials, experimental systems and methods used in many studies. Here, indicate whether each material, system or method listed is relevant to your study. If you are not sure if a list item applies to your research, read the appropriate section before selecting a response.

Materials & experimental systems

n/a	Involved in the study	
☒	☐	Antibodies
☒	☐	Eukaryotic cell lines
☒	☐	Palaeontology and archaeology
☒	☐	Animals and other organisms
☒	☐	Clinical data
☒	☐	Dual use research of concern
☒	☐	Plants

Methods

n/a	Involved in the study	
☒	☐	ChIP-seq
☒	☐	Flow cytometry
☒	☐	MRI-based neuroimaging
